# Vitamin D, acute respiratory infections, and Covid-19: The curse of small-size randomised trials. A critical review with meta-analysis of randomised trials

**DOI:** 10.1371/journal.pone.0303316

**Published:** 2025-01-14

**Authors:** Philippe Autier, Giulia Doi, Patrick Mullie, Patrick Vankrunkelsven, Oriana D’Ecclesiis, Sara Gandini

**Affiliations:** 1 International Prevention Research Institute (iPRI), Bat. L’Américain, Dardilly, France; 2 Department of Experimental Oncology, European Institute of Oncology, IRCCS, Milano, Italy; 3 Academic Centre for General Practice, Department of Public Health and Primary Care, Catholic University Leuven, Leuven, Belgium; 4 Belgian Centre for Evidence-Based Medicine, Cochrane Belgium, Leuven, Belgium; 5 DG H&WB, Queen Elisabeth Barracks, Belgian Defence, Evere, Belgium; University of Oxford, UNITED KINGDOM OF GREAT BRITAIN AND NORTHERN IRELAND

## Abstract

**Background:**

Randomised trials conducted from 2006 to 2021 indicated that vitamin D supplementation (VDS) was able to prevent severe COVID-19 and acute respiratory infections (ARI). However, larger randomised trials published in 2022 did not confirm the health benefits of VDS in COVID-19 patients.

**Objective:**

To examine through a systematic review with meta-analysis the characteristics of randomised trials on VDS to COVID-19 patients and admission to intensive care unit (ICU), and of randomised trials on VDS for the prevention of ARI.

**Method:**

A systematic search retrieved randomised trials on VDS to COVID-19 patients and admission to ICU. Data on VDS and ARI were extracted from the meta-analysis of Jolliffe et al. 2021. Groups were formed including trials with total numbers of patients below or above the median size of all trials. The associations between VDS vs no VDS, and admission to ICU were evaluated using random-effects models from which summary odds ratios (SOR) and 95% confidence intervals (CI) were obtained. Meta-analyses were done for all trials and for each group of trials, which allowed testing a possible effect modification of trial size. Publication bias was assessed using the Louis-Furuya-Kanaruori (LFK) index (no bias if index between -1 and +1) and the trim and fill method.

**Results:**

Nine trials on VDS for preventing admission to ICU were identified, including 50 to 548 patients. The summary odds ratio (SOR) was 0.61 (95% CI: 0.39–0.95) for all trials, 0.34 (0.13–0.93) for trials including 50 to <106 patients and 0.88 (0.62–1.24) for trials including 106 to 548 patients (interaction p = 0.04). The LFK index was -3.79, and after trim and fill, the SOR was 0.80 (0.40–1.61). The SOR for the 37 trials on VDS for ARI prevention included 25 to 16,000 patients. The SOR was 0.92 (0.86–0.99) for all trials, 0.69 (0.57–0.83) for trials including 25 to <248 patients and 0.98 (0.94–1.03) for trials including 248 to 16,000 patients (interaction p = 0.0001). The LFK index was -3.11, and after trim and fill, the SOR was 0.96 (0.88–1.05).

**Conclusion:**

Strong publication bias affected small randomised trials on VDS for the prevention of severe COVID-19 and of ARI. Systematic reviews should beware of small-size randomised trials that generally exaggerate health benefits.

## Introduction

When the COVID-19 pandemic struck the World in 2019–2021, many thought that vitamin D supplementation (VDS) was an option for reducing the risk of COVID-19 and progression to severe disease [1–3]. The VDS option was nurtured by a wealth of data. First, laboratory studies have documented the involvement of physiologically active forms of vitamin D in immunological mechanisms dealing with infectious agents [4–7]. Second, the serum concentration of 25-hydroxyvitamin D (hereafter s-25OHD) is usually considered reflecting individual vitamin D status. Numerous observational studies done before and after the pandemic onset found first that patients with low s-25OHD were at higher risk of severe acute respiratory infections (ARI) and severe COVID-19 [8–10], and second that COVID-19 patients taking VDS had less severe disease course [11]. Third, a meta-analysis of aggregated data from 37 trials conducted between 2006 and 2019 found that VDS was associated with a 8% (95% confidence interval [CI]: 1% to 14%) reduction in the risk of ARI [12]. Fourth, randomised trials published in 2020 and 2021 obtained results compatible with a favourable effect of VDS on COVID-19 outcomes [13].

However, the promises of the VDS option were tempered by the publication in 2022 of large size randomised trials that found no effect of VDS on the incidence and severity of COVID-19 [14–16]. In addition, six Mendelian randomisation studies examined the risk of incidence and severity of COVID-19 in patients whose s-25OHD is lower than average because of their genetic background. Patients with naturally low s-25OHD had no greater risk of COVID-19 incidence or severity than patients with higher s-25OHD [17–22].

Systematic reviews play a pivotal role in the development of clinical practice guidelines and the formulation of healthcare policies, serving as a robust foundation for setting standards of care [23]. Therefore the disillusionment caused by Mendelian and large-size randomised trials called for a review of the thread of data which resulted in considering VDS as an option for the treatment of COVID-19. This article is a systematic review with meta-analysis of randomised trials on VDS for the prevention of severe COVID-19 in patients infected with the SARS-CoV-2 virus. This article also revisited the meta-analysis of randomised trials of VDS done before 2020 for the prevention of ARI [12].

## Methods

### Literature search

This review was not registered. A scoping review of the literature end of 2022 showed that the most robust outcome indicating severe COVID-19 disease was the admission to intensive care unit (ICU). Other possible outcomes were less robust (e.g. admission to hospital), or possibly influenced by subjectivity (e.g. self-reported dyspnoea, resolution of symptoms, duration of hospitalisation).

In October 2022, PA and PM performed a systematic search restricted to PubMed of randomised trials published since January 2000 on VDS and outcomes of COVID-19 patients aged 18 years or more. The search was done in two successive steps (S1 File). We also searched references cited in review articles.

Studies were selected if (i) they reported on randomised trials that compared rates of admission to ICU or COVID-19 patients receiving VDS with patients not receiving VDS, (ii) VDS was the unique intervention, (iii) COVID-19 patients enrolled in trials were not taking VDS before trial enrolment, (iv) comparison groups included COVID-19 patients who did not receive any type of VDS during trial courses, and (v) published in English language. All forms of vitamin D were considered (ergocalciferol (vitamin D2), cholecalciferol (vitamin D3), calcifediol (25-hydroxycholecalciferol), calcitriol (1α,25-dihydroxycholecalciferol)). The selection of article was performed by all authors, with disagreements resolved via consensus.

### Data extraction

Key study characteristics and data of selected studies were first extracted by PA and PM, and then independently verified by GD and OD. Disagreements were resolved via consensus.

Extracted information were first author, publication year, patient numbers by randomisation group, intervention, comparison groups (i.e. whether the VDS intervention was tested against a placebo, or no placebo), and numbers of patients with outcome by randomisation group. Key data on patient numbers and outcomes (i.e. odds ratios [OR] and 95% CI) included in the meta-analysis of Jolliffe et al. 2021 [12] were extracted by PA and PM. There was no missing data.

### Risk of bias assessment

For trials on VDS in COVID-19 patients and admission to ICU, we assessed seven sources of bias following items of the Cochrane’s risk of bias (RoB-1) tool [24]:

- patient selection and randomisation procedure,
- placebo control, yes or no,
- blinding to evaluate the risk of performance bias due to knowledge of the allocated interventions by participants and personnel during the study,
- allocation concealment to evaluate the risk of selection bias (biased allocation to interventions) due to inadequate concealment of allocations prior to assignment,
- outcome assessment to assess the risk of reporting bias due to selective outcome reporting.
- analysis to evaluate the risk of attrition bias due to amount, nature or handling of incomplete outcome data.
- other sources of bias.

In order to illustrate the RoB results, we used a bar plot of the distribution of risk-of-bias judgments within each bias domain and a "traffic light" plot [25].

### Statistical analysis

For trials in COVID-19 patients, risks of admission to ICU were computed as odds ratios with 95% CI following a per-protocol approach. Hence, statistical analyses were done using data from patients who took the VDS or the placebo (when applicable) according to trial protocols.

Standard errors (SE) of odds ratios (OR) were derived from the equation SQRT(1/a+1/b+1/c+1/d), where a and b are the numbers of patients admitted to ICU in the VDS and in the comparison group, respectively, and c and d are the numbers of patients not admitted to ICU in the VDS and in the comparison group, respectively.

For the meta-analysis on VDS of COVID-19 patients and admission in ICU, we used random-effects models to calculate summary odds ratios and 95% CI of admission to ICU of COVID-19 patients.

Random-effects models necessitate the estimation of the between-study variance τ^2^. Various methods exist for computing an estimator of the between-study variance τ^2^ and for estimating its confidence interval. Mathematical simulations have shown that the widely used Der Simonian-Laird estimator is known to be negatively biased in scenarios with small studies and in scenarios with a rare binary outcome [26].

Because most randomised trials found in the systematic search were of small and medium size, i.e. from about 40 to 400 patients enrolled, we opted to use the REML (Restricted Maximum Likelihood) estimator, and the REML estimator combined with the HKSJ (Hartung-Knapp-Sidik-Jonkman) method for estimating the 95% CI [27, 28].

For the meta-analysis on VDS for ARI prevention [12], we computed pooled estimate applying the Der Simonian-Laird method in order to reproduce the original analysis.

Then for both meta-analyses, statistical heterogeneity was evaluated through the I^2^ index: a value <50% was considered indicative of low or moderate between-study heterogeneity [29]. To investigate the between-study heterogeneity and the stability of the pooled estimate, we carried out a sensitivity analysis by excluding from the meta-analysis one of the trials at a time.

We evaluated the presence of possible publication bias, using Egger’s regression test, and the Makaskill test. We also used the method proposed by Furuya-Kanamori et al. [30], which displays in a Doi plot the weight of each trial (i.e. the sample size of each trial relative to the size of all trials combined, expressed as a Z-score) against the natural logarithm of odds ratios reported by trials. The Louis-Furuya-Kanaruori (LFK) index derived from the Doi plot provides a quantitative estimate of the asymmetry of odds ratios included in the meta-analysis. A LFK index of zero indicates an absence of asymmetry. Asymmetry, and thus the possibility of publication bias, is suggested by a LFK index less than -1 (studies not in favour of VDS less likely to be published) or greater than +1 (studies in favour of VDS less likely to be published).

We performed an exploratory analysis of publication bias using the trim and fill method [31], which provides summary odds ratios adjusted for publication bias.

We studied the effect of the sample size through a subgroup analysis, by dividing the trials based on their sample sizes (lower or greater than the median), and including in our statistical models the trial size as a possible effect modifier.

All reported p-values were two sided and p<0.05 was considered statistically significant. Meta-analyses were carried out by using the R-Studio software (R version 4.1.1).

## Results

### VDS to COVID-19 patients and admission to ICU

**Fig 1:**
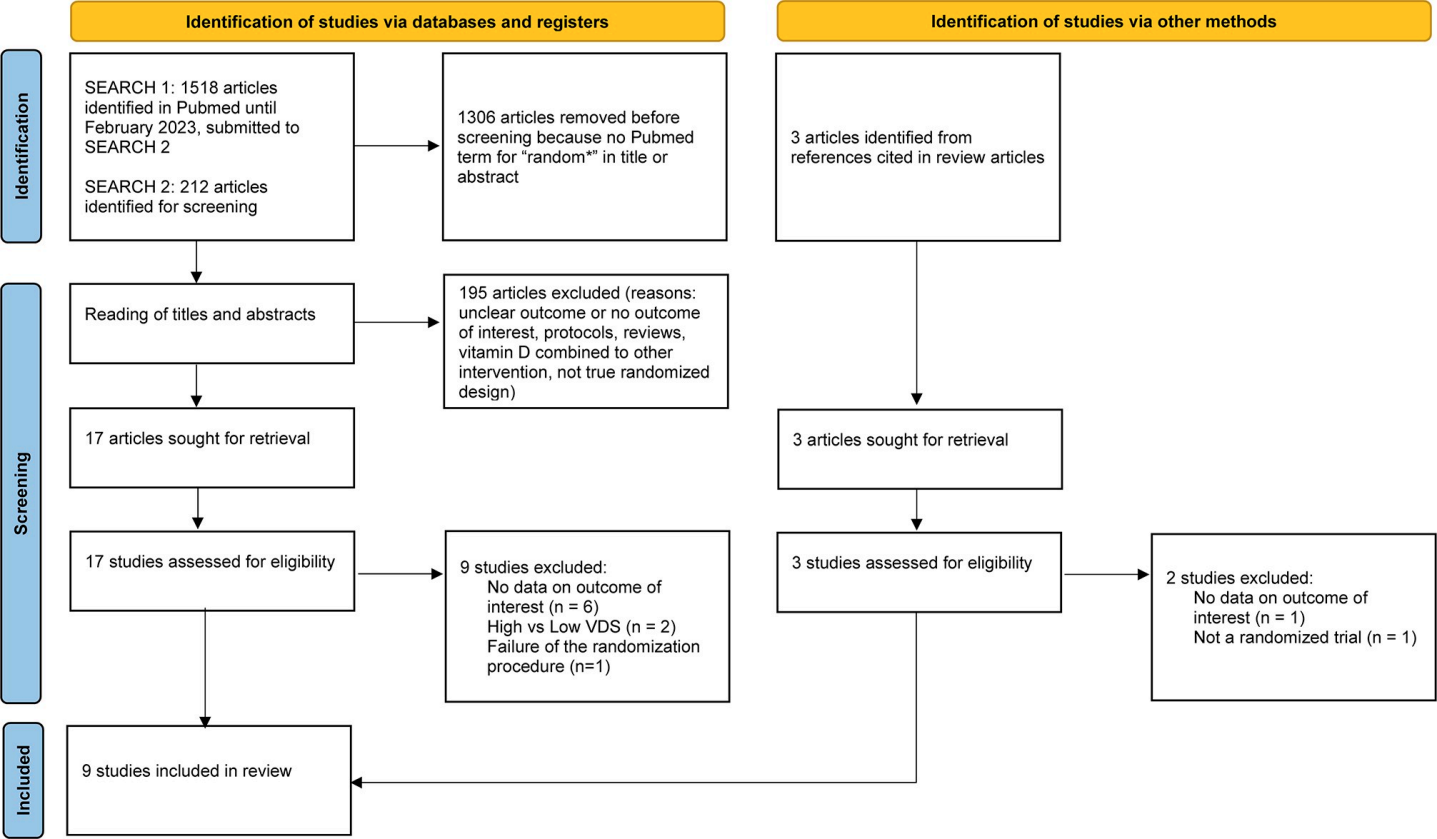
PRISMA 2020 literature search flow diagram.

The literature search is depicted in Fig 1. Three articles [32–34] were found during hand search of review articles. After full article reading, nine articles [16, 32, 35–41] were selected for review and 11 articles were excluded (S2 Table). Reasons for exclusion were: high vs. low dose VDS [42, 43], no specific numbers of patients who were admitted in ICU [14, 15, 34, 44–47], failure of randomisation [33], and not a randomised trial [48].

Characteristics of the nine selected randomised trials are summarized in Table 1. Data extracted for the meta-analysis are displayed in S3 Table.

**Table 1:** Randomised trials on vitamin D supplementation of COVID-19 patients and admission in ICU.

First author, year	VDS intervention	Placebo in control group	Total No. randomised*	No. patients		Patients with outcome		Risk estimate*	95%CI
				VDS group	Control group	VDS group	Control group		
Entrenas-Castillo, 2020	0.532 mg calcifediol; day 1, 3, 7	No, OL	76	50	26	1	13	0.02	0.00-0.17
Maghbooli, 2021	25 μg/d calcifediol; 60 days	Yes	106	53	53	6	10	0.55	0.18-1.64
Murai, 2021	5,000 μg cholecalciferol; single IV bolus	Yes	235	117	118	19	25	0.72	0.37-1.40
De Niet, 2022	4 days with 625 μg/d cholecalciferol and 6 weeks with 625 μg/w	Yes	43	21	22	2	5	0.36	0.06-2.09
Elamir, 2022	0.5 μg/d calcitriol; 14 days	No, OL	50	25	25	5	8	0.53	0.15-1.93
Karonova, 2022	1,250 μg cholecalciferol on days 1 and 8	No, OL	110	56	54	0	3	0.13	0.01-2.58
Lakkireddy, 2022	1,500 μg/d cholecalciferol, 8 days for subjects with body mass index (BMI) of 18-25 kg/m^2^ and 10 days for subjects with BMI >25 kg.m^2^	No, OL	87	44	43	4	5	0.76	0.19-3.04
Cannata, 2022	2,500 μg cholecalciferol; single IV bolus	No, OL	543	274	269	47	44	1.06	0.68-1.66
Mariani, 2022	12,500 μg cholecalciferol; single IV bolus	Yes	218	115	103	9	11	0.71	0.28-1.79

Data extracted from 12 to 16 of September 2022; CI: confidence interval; VDS: vitamin D supplementation; ICU: intensive care unit; ITT: intent-to-treat; OL: open label

*Odds ratio from per protocol approach.

The vitamin D compound were cholecalciferol (5 trials), calcifediol (2 trials), or calcitriol (1 trial). VDS dosages and regimens were variable with daily intake in six trials and bolus administration in three trials. Four trials were placebo controlled and five trials were open-label. The numbers of COVID-19 patients enrolled in trials ranged from 50 to 548. The odds ratios for admission in ICU ranged from 0.02 to 1.06, with wide 95% CI. The risk of bias analysis is detailed in S1 Fig. Five trials had a low risk of bias, and four had a high risk of bias due to lack of placebo groups.

**Fig 2:**
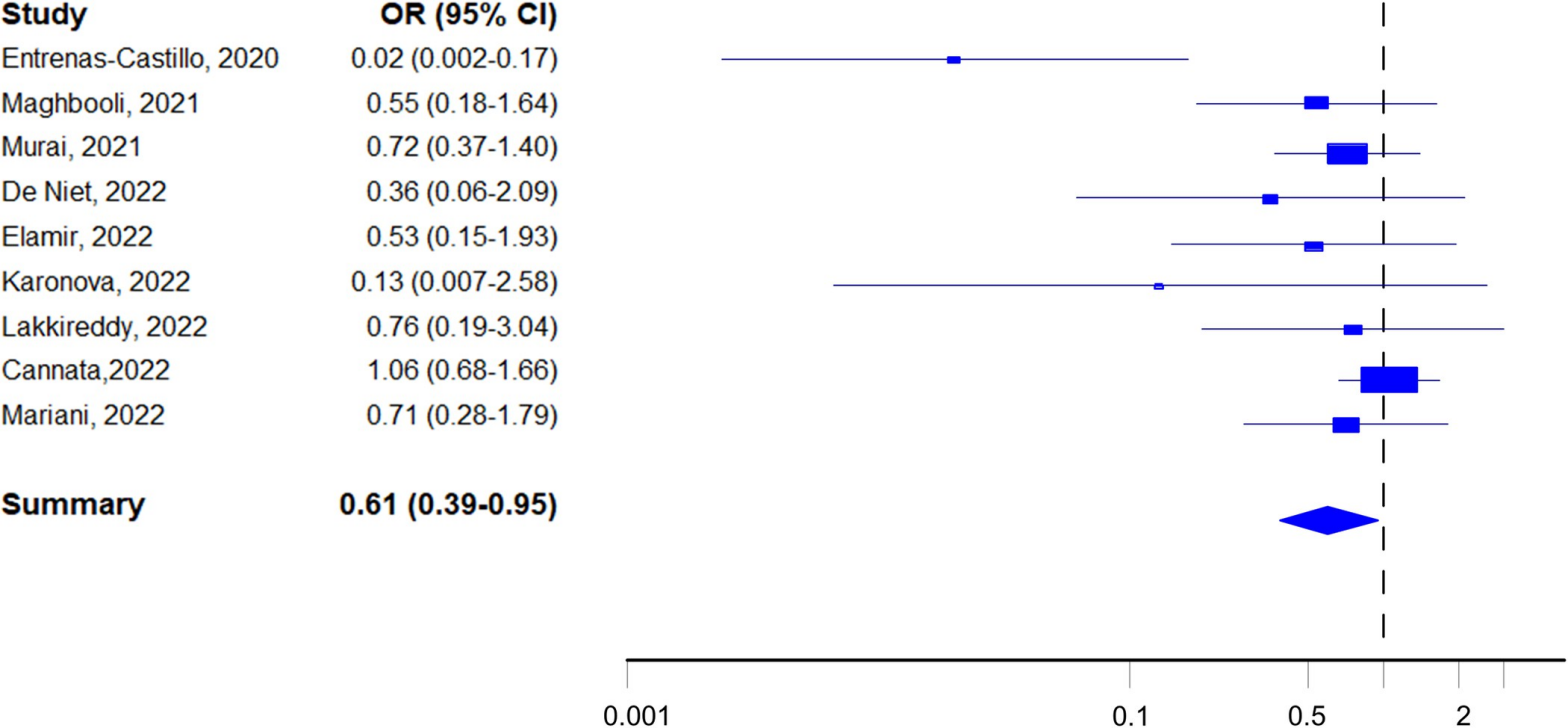
Forest plot of randomised trials on VDS of COVID-19 patients and admission to ICU.

The meta-analysis of the nine trials based on the RMLE estimator resulted in a summary odds ratio of 0.61 (95% CI: 0.39–0.95) (Fig 2, Table 2), with low heterogeneity (I^2^ = 35%), suggesting that overall COVID-19 patients taking VDS would have a substantially decreased risk of admission to ICU.

**Table 2:** Meta-analysis of randomised trials on VDS of COVID-19 patients and admission in ICU.

**Restricted Maximum Likelihood Estimator**			
9 trials			
Summary risk estimate	SOR (95% CI)	0.61	(0.39–0.95)*
Heterogeneity	I^2^	35%	
Publication bias	Egger test	p = 0.001	
	Modified Macaskill test	p = 0.03	
Summary risk estimate after trim & fill	OR (95% CI)	0.80	(0.40–1.61)
8 trials without Entrenas-Castillo et al. 2020			
Summary risk estimate	SOR (95% CI)	0.78	(0.57–1.08)
Heterogeneity	I^2^	4%	
5 trials which included less than 106 patients			
Summary risk estimate	SOR (95% CI)	0.34	(0.13–0.93)
4 trials which included 106 patients or more			
Summary risk estimate	SOR (95% CI)	0.88	(0.62–1.24)
Interaction between randomisation group and trial size §		p = 0.04	
**Restricted Maximum Likelihood Estimator plus Hartung-Knapp-Sidik-Jonkman for 95% CI**			
9 trials			
Summary risk estimate	SOR (95% CI)	0.61	(0.32–1.16)
Heterogeneity	I^2^	35%	
Publication bias	Egger test	p = 0.0086	
	Modified Macaskill test	p = 0.1195	
Summary risk estimate after trim & fill	OR (95% CI)	0.80	(0.33–1.97)
8 trials without Entrenas-Castillo et al. 2020			
Summary risk estimate	SOR (95% CI)	0.78	(0.58–1.06)
Heterogeneity	I^2^	3.7%	
5 trials which included less than 106 patients			
Summary risk estimate	SOR (95% CI)	0.34	(0.07–1.72)
4 trials which included 106 patients or more			
Summary risk estimate	SOR (95% CI)	0.88	(0.51–1.50)
Interaction between randomisation group and trial size §		p = 0.16	

ICU: intensive care unit; OR: odds ratio; SOR: summary odds ratio; VDS: vitamin D supplementation

*0.05>p>0.001

§ Interaction term combining randomisation group (VDS vs comparison) to trial size (<106 vs ≥106 patients)

**Fig 3:**
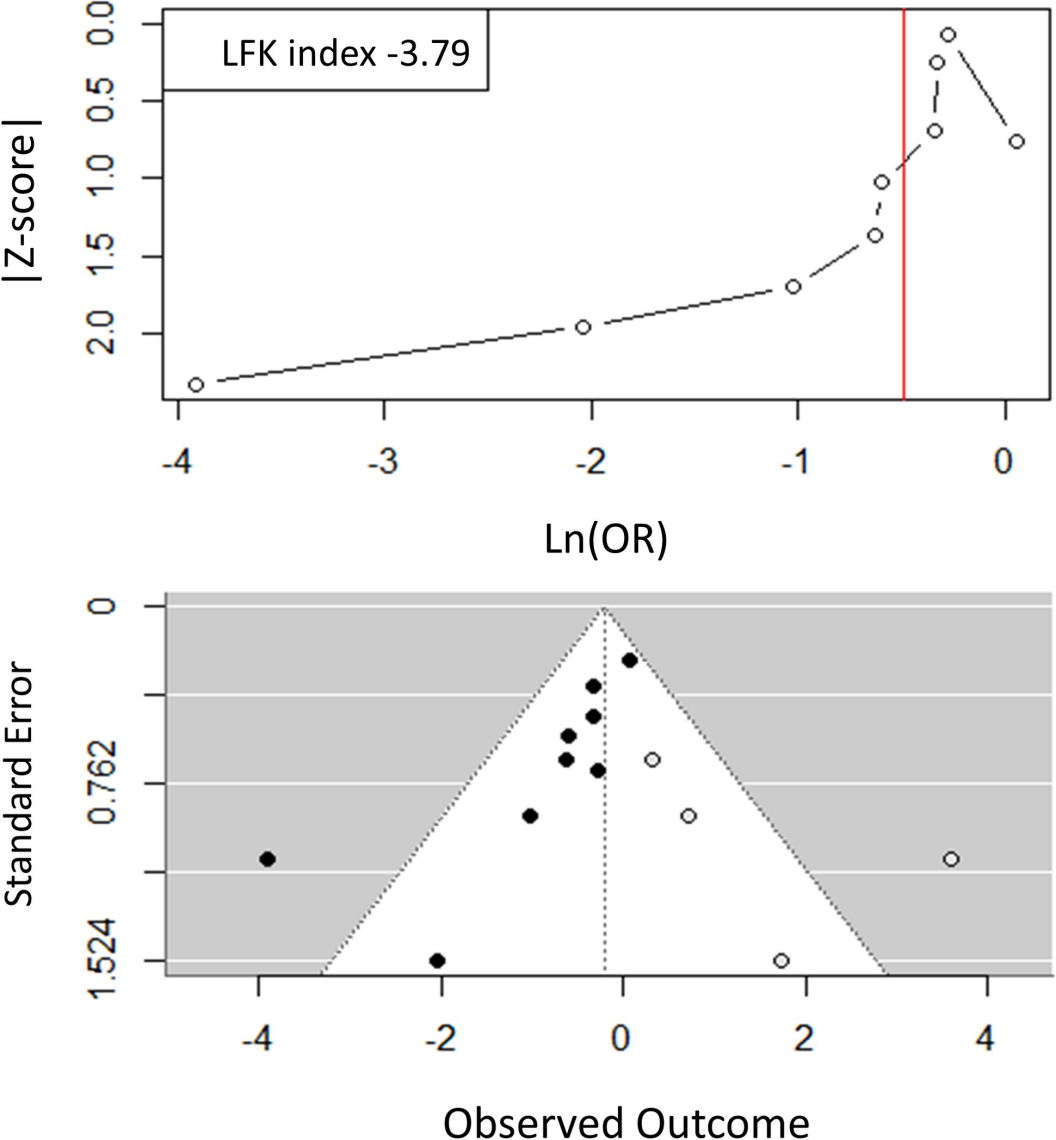
Doi plot (a) and funnel plot after trim and fill correction (b) for randomised trials on VDS and admission to ICU.

The Egger and the modified Macaskill tests indicated significant publication bias (Table 2). The display of odds ratios from the nine trials in a Doi plot (Fig 3A) showed a distribution markedly stretched to the left, witnessing a negative publication bias, i.e. trials not favouring a reduction of admissions to ICU associated with VDS were less likely to be published than trials favouring a reduction of admissions to ICU associated with VDS. Of note, the smaller the trial size (i.e. the higher the Z-score), the greater the risk reductions, i.e. ln(OR) moving towards negative values far from zero.

The LFK index of -3.79, well below -1.0, quantifies the strong imbalance by which trials in favour of VDS had a much greater probability to be published than trials in favour of VDS.

The trim and fill method resulted in the addition of four open circles on the funnel plot (Fig 3B). These open circles are imputations of results of four hypothetical smaller-size trials not in favour of VDS that should have been published for completely excluding the possibility of publication bias. After application of the trim and fill method, the summary odds ratio increased to 0.80 (95% CI: 0.40–1.61), which was no longer significant.

Exclusion of one trial at a time from the meta-analysis showed that exclusion of Entrenas-Castillo et al. 2020 [35] of the meta-analysis ended up in a non-significant summary odds ratio of 0.78, with a drastic drop in heterogeneity between results of the eight other trials (I^2^ = 4%).

After stratification of trials according to numbers of patients enrolled (Table 2), the summary odds ratios were 0.34 (95% CI: 0.13–0.93) for the five trials including 50 to 105 COVID-19 patients, and 0.88 (95% CI: 0.62–1.24) for the four trials including 106 to 548 patients. The interaction between random group allocation and trial size had a p-value of 0.04, suggesting that sample size was an effect modifier.

Use of the RMLE estimator with Hartung-Knapp-Sidik-Jonkman (HKSJ) method for the 95% CI resulted in a summary odds ratio of 0.61 (95% CI: 0.32–1.16). If the summary odds ratio remained the same, the 95% CI was wider, from 0.56 (= 0.95 minus 0.39) when the RMLE estimator only was used to 0.72 (= 1.16 minus 0.32) when the RMLE estimator and the HKSJ methods were used. Because of the wider 95% CI, the risk of admission to ICU was no longer significant. All other meta-analysis results remained equivalent or close to results when the RMLE estimator only was used, but owing to the larger confidence interval, the interaction between random group allocation and trial size was no longer significant.

### VDS for ARI prevention

The 37 trials on VDS for the prevention of ARI included in the publication of Jolliffe et al. 2021 [12] enrolled 25 to 16,000 participants (median of 247). The meta-analysis of the 37 trials resulted in a summary odds ratio of 0.92 (95% CI: 0.86–0.99), suggesting a beneficial effect of VDS on the risk of ARI. But the Egger and the modified Macaskill tests provided evidence for significant publication bias (Table 3).

**Table 3:** Meta-analysis of randomised trials on VDS of the prevention of ARI and admission in ICU.

**Restricted Maximum Likelihood Estimator**			
37 trials			
SRE	OR (95% CI)	0.92	(0.86–0.99)
Heterogeneity	I2	36%	
Publication bias	Egger test	p = 0.007	
	Modified Macaskill test	p = 0.0004	
After trim & fill	OR (95% CI)	0.96	(0.88–1.05)
19 trials which included less than 248 patients			
SRE	OR (95% CI)	0.69	(0.57–0.83)
18 trials which included 248 patients or more			
SRE	OR (95% CI)	0.98	(0.94–1.03)
Interaction between randomisation group and trial size *		p = 0.0001	

ARI: acute respiratory infection; VDS: vitamin D supplementation; OR: odds ratio.

SRE: summary risk estimate

* Interaction term combining randomisation group (VDS vs comparison) to trial size (<248 vs ≥248 patients)

**Fig 4:**
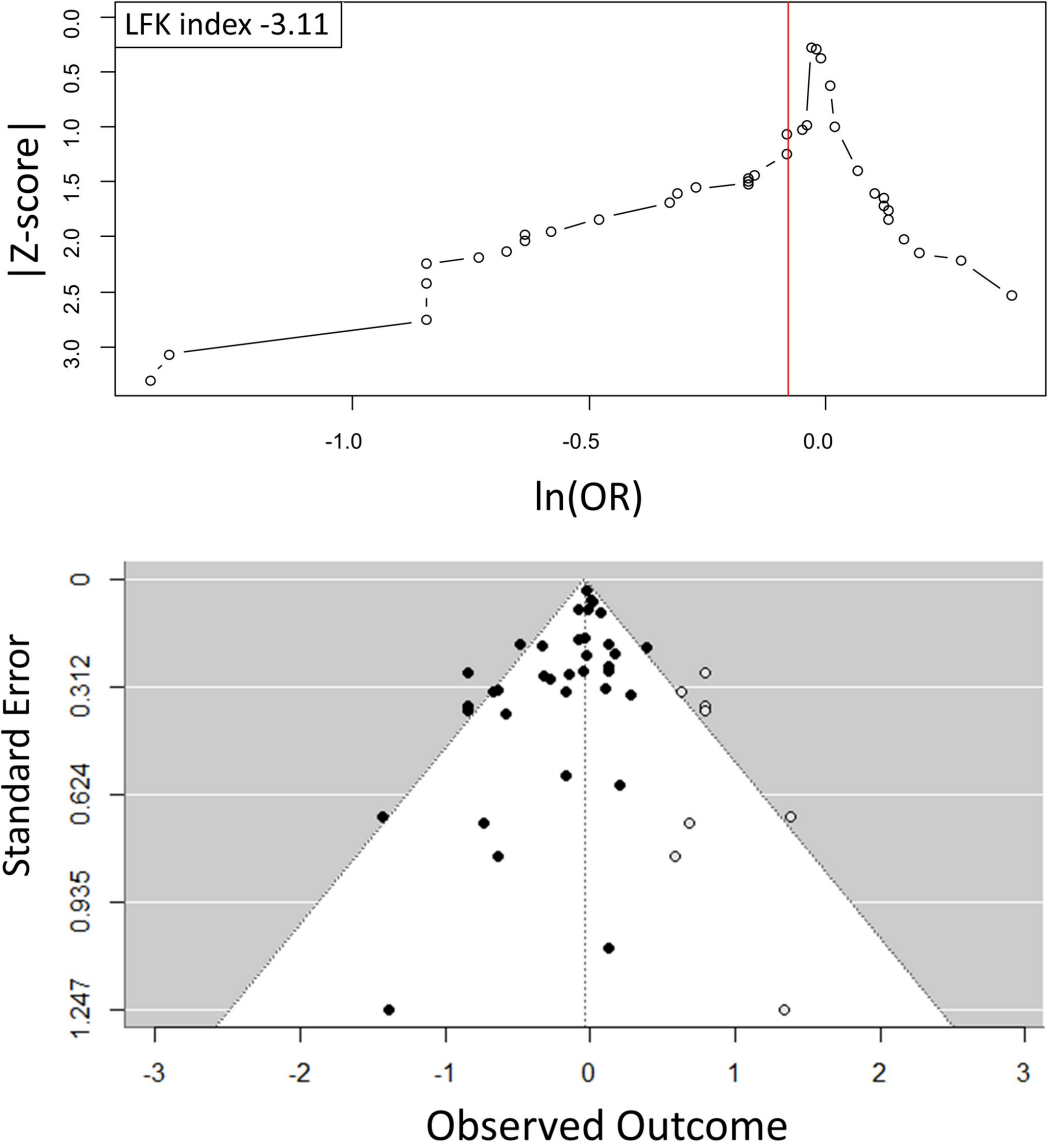
Doi plot (a) and funnel plot after trim and fill correction (b) for randomised trials on VDS and occurrence of ARI.

The display of odds ratios from the 37 trials in a Doi plot (Fig 4A) showed a distribution markedly stretched to the left, witnessing a negative publication bias, i.e. trials not favouring a reduction of ARI associated with VDS were less likely to be published than trials favouring a reduction of ARI associated with VDS. The smaller the trial size (i.e. the higher the Z-Score), the greater the reduction of the risk of ARI, i.e. ln(OR) moving towards negative values far from zero.

The LFK index of -3.11, well below -1.0, quantifies the strong imbalance by which trials in favour of VDS had a much greater probability to be published than trials not in favour of VDS.

The trim and fill method resulted in the addition of eight open-circle points on the funnel plot (Fig 4B). These open-circle points are imputed results of hypothetical smaller-size trials not in favour of VDS that should have been published for completely excluding the possibility of publication bias. After application of the trim and fill method, the summary odds ratio increased to 0.96 (95% CI: 0.88–1.95), which was no longer significant.

The meta-analysis of the 19 trials including 25 to 247 patients obtained a summary odds ratio of 0.69 (95% CI: 0.57–0.83) suggesting marked protective effect of VDS. Instead, the meta-analysis of the 18 trials including 250 to 16,000 patients or more resulted in a summary odds ratio of 0.98 (95% CI: 0.94–1.03) indicating no protective effect of VDS. The interaction between random group allocation and trial size had a p-value of 0.0001, suggesting that sample size was an effect modifier.

## Discussion

Our review on VDS for COVID-19 patients and the risk of admission to ICU, and on VDS for the prevention of ARI shows that the majority of small-size randomised trials obtained results in sharp contrast with results of medium-to-large-size randomised trials. Results of medium-size randomised trials in the COVID-19 setting are in agreement with the six aforementioned Mendelian randomisation studies that examined the risk of incidence and severity of COVID-19 according to s-25OHD. They also agree with the negative results of large-size randomised trials conducted before 2020 on high-dose VDS for critically ill, vitamin D deficient patients admitted in intensive care unit [49, 50].

Hence publication bias was probably present, meaning that a number of small-size trials that obtained odds ratio not in favour of VDS were not published because either authors did not consider that their work was worth being submitted to medical journals, or because submissions were turned down by medical journals.

When the COVID-19 pandemic expanded, many new open-source resources were created for rapid dissemination of research findings, with the consequence of more lenience on the quality of research [51].

Jolliffe et al. 2021 [12] considered that virtually all 37 trials were at low risk of bias, which is surprising in view of the contrasting results between small-size and large-size trials. Although in the original publication, a funnel plot highlighted a left-sided asymmetry with Egger’s test having a p-value of 0.007, the authors considered that these results “might reflect heterogeneity of effect across trials, or publication bias arising from omission of small trials showing non-protective effects of vitamin D supplementation from the meta-analysis”.

The interpretation of small-size trial effects is notoriously tricky. Overrepresentation of small-size trials with substantial effect on outcome can be due to publication bias and to other reasons like poorer quality of small-size trials compared to large-size trials, or selective reporting of results [52–55].

Systematic reviews with meta-analyses of randomised trials are considered the optimal methodology for evaluating treatment efficacy. However, our study shows that, regardless of being the result of publication bias or poor-quality research, small-size randomised trials can lead to overestimate the efficacy of interventions [55, 56]. The undesirable effects of small-size trials have been reported for a variety of therapeutic areas [57], including critical care [58].

### Study limitations

Our study has several limitations. The study unfolded with the uncovering of the effects of trial sizes on the associations between VDS and acute respiratory infections or COVID-19 severity. In this regard, the study did not follow a protocol written before looking at the data. We reused data from Jolliffe et al. 2021 without a literature search for more recent publication on randomised trials testing VDS for acute respiratory infections. The literature search on VDS for COVID-19 was restricted to the PubMed and reference lists of review articles published in 2022. The selection criteria we adopted led to the exclusion of 11 randomised trials, and we did not explore the grey literature, considering that in this particular setting, the published literature had more influence on clinical practice. However, we believe that more extensive literature searches were not likely to find other publications relevant to this work.

Tests for publication bias have known limitations, including the LFK index [59]. Therefore, our analyses used four different tests, all of which confirmed the likelihood of publication bias.

### Low vitamin D status and reverse causation

The negative results of medium-size trials on VDS are in striking contrast with the common observation that low s-25OHD is associated with more severe COVID-19 and death. This contrast underscores that low s-25OHD would have no detrimental role in COVID-19 but would rather be the consequence of two sets of factors (i.e. reverse causation). First, s-25OHD seems to behave like a negative surface reactant whose serum concentration fall during inflammatory states, just like albumin, transferrin and other compounds [44, 60–63]. Second, patient’s characteristics and medical history known to precipitate severe COVID-19, like age, diabetes, obesity, frailty, cardiovascular conditions, are also known risk factors for low s-25OHD [64].

The associations between low s-25OHD and COVID-19 severity echoes the many observational studies showing that patients with low s-25OHD are at higher risk of acute and chronic disease, including cardiovascular diseases, diabetes, and fractures, and of premature death [64, 65]. However, randomised trials and their meta-analyses have failed to confirm that VDS could prevent or treat any of these diseases and prolong life-expectancy [66–69].

## Conclusions

Our review showed that the alleged protection of VDS against severe COVID-19 was the consequence of the of small-size randomised trials published before and in the early stages of the COVID-19 pandemic. If in 2022, it was recognised that VDS brought no benefit to COVID-19 patients [70, 71], mechanistic data, observational studies and small-size randomised trials done before have contributed to treating many COVID-19 patients with VDS. The expectation is that in the future, a same thread of laboratory and observational studies, combined with small size randomised trials would not lead to the adoption of poorly effective but unsafe preventive or therapeutic interventions, until larger randomised trials, that take more time to deliver results, would discourage their use.

## Supporting information

S1 File Keywords used for literature search in PubMed and PRISMA flow chart. (DOCX)

S1 Fig Risk of bias in randomised trials on VDS of COVID-19 patients and admission to ICU. (DOCX)

S1 Table Studies identified during the literature search, with inclusions and exclusions. (DOCX)

S2 Table Data extracted for the meta-analysis. (DOCX)

S3 Table PRISMA 2020 checklist. (DOCX)
