# Supplementary material for: Vitamin D, acute respiratory infections, and Covid-19: The curse of small-size randomised trials. A critical review with meta-analysis of randomised trials
Source: PLoS One. 2025 Jan 14;20(1):e0303316. doi: 10.1371/journal.pone.0303316 (PMC11731873; doi:10.1371/journal.pone.0303316)
Supplement: S1 File — (DOCX) [file pone.0303316.s001.docx]

**S1 File. Keywords used for literature search in PubMed**

A first list of articles was identified following Search 1 keywords. A second set of keywords under Search 2 was applied on the first list. The search was until February 2023.

**Search 1**

**Vitamin D supplementation AND COVID-19**

("vitamin d"[MeSH Terms] OR "ergocalciferols"[MeSH Terms] OR “vitamin d”[Text Word] OR ("calcitriol"[MeSH Terms] OR calcitriol[Text Word]) OR ("calcifediol"[MeSH Terms] OR calcifediol[Text Word]) OR ("cholecalciferol"[MeSH Terms] OR cholecalciferol[Text Word])) AND ("COVID-19"[All Fields] OR "COVID-19"[MeSH Terms] OR "COVID-19 Vaccines"[All Fields] OR "COVID-19 Vaccines"[MeSH Terms] OR "COVID-19 serotherapy"[All Fields] OR "COVID-19 serotherapy"[All Fields] OR "COVID-19 Nucleic Acid Testing"[All Fields] OR "covid-19 nucleic acid testing"[MeSH Terms] OR "COVID-19 Serological Testing"[All Fields] OR "covid-19 serological testing"[MeSH Terms] OR "COVID-19 Testing"[All Fields] OR "covid-19 testing"[MeSH Terms] OR "SARS-CoV-2"[All Fields] OR "sars-cov-2"[MeSH Terms] OR "Severe Acute Respiratory Syndrome Coronavirus 2"[All Fields] OR "NCOV"[All Fields] OR "2019 NCOV"[All Fields])

1518 articles

**Search 2**

**Vitamin D supplementation AND COVID-19 AND Random* (Title/Abstract)**

("vitamin d"[MeSH Terms] OR "ergocalciferols"[MeSH Terms] OR "vitamin d"[Text Word] OR ("calcitriol"[MeSH Terms] OR calcitriol[Text Word]) OR ("calcifediol"[MeSH Terms] OR calcifediol[Text Word]) OR ("cholecalciferol"[MeSH Terms] OR cholecalciferol[Text Word])) AND ("COVID-19"[All Fields] OR "COVID-19"[MeSH Terms] OR "COVID-19 Vaccines"[All Fields] OR "COVID-19 Vaccines"[MeSH Terms] OR "COVID-19 serotherapy"[All Fields] OR "COVID-19 serotherapy"[All Fields] OR "COVID-19 Nucleic Acid Testing"[All Fields] OR "covid-19 nucleic acid testing"[MeSH Terms] OR "COVID-19 Serological Testing"[All Fields] OR "covid-19 serological testing"[MeSH Terms] OR "COVID-19 Testing"[All Fields] OR "covid-19 testing"[MeSH Terms] OR "SARS-CoV-2"[All Fields] OR "sars-cov-2"[MeSH Terms] OR "Severe Acute Respiratory Syndrome Coronavirus 2"[All Fields] OR "NCOV"[All Fields] OR "2019 NCOV"[All Fields]) AND random*[Title/Abstract]

212 articles
