# Supplementary figures and images for: Vitamin D, acute respiratory infections, and Covid-19: The curse of small-size randomised trials. A critical review with meta-analysis of randomised trials
Source: PLoS One. 2025 Jan 14;20(1):e0303316. doi: 10.1371/journal.pone.0303316 (PMC11731873; doi:10.1371/journal.pone.0303316)

**S1 Fig. Risk of bias in randomised trials on VDS of COVID-19 patients and admission to ICU**


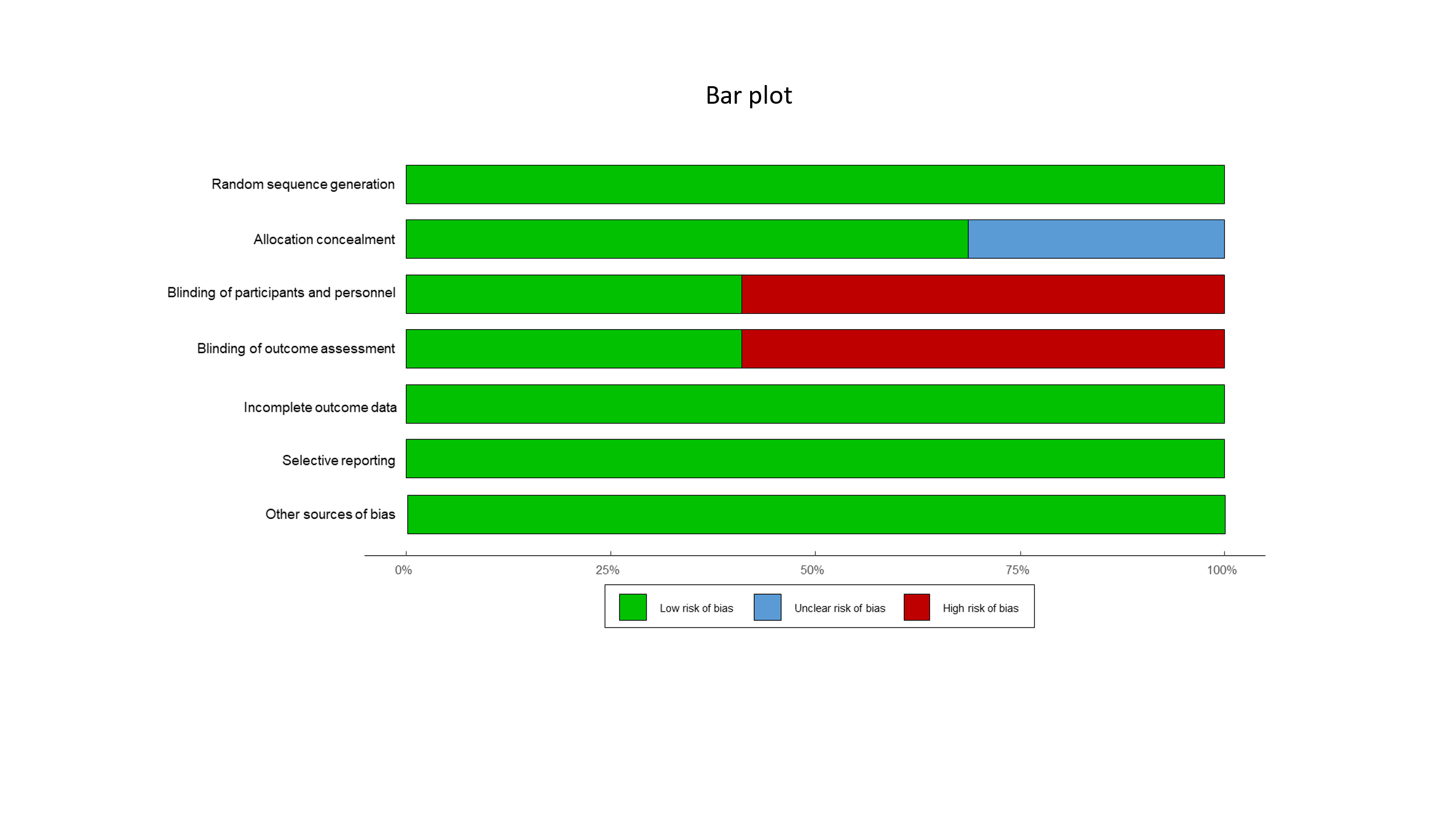


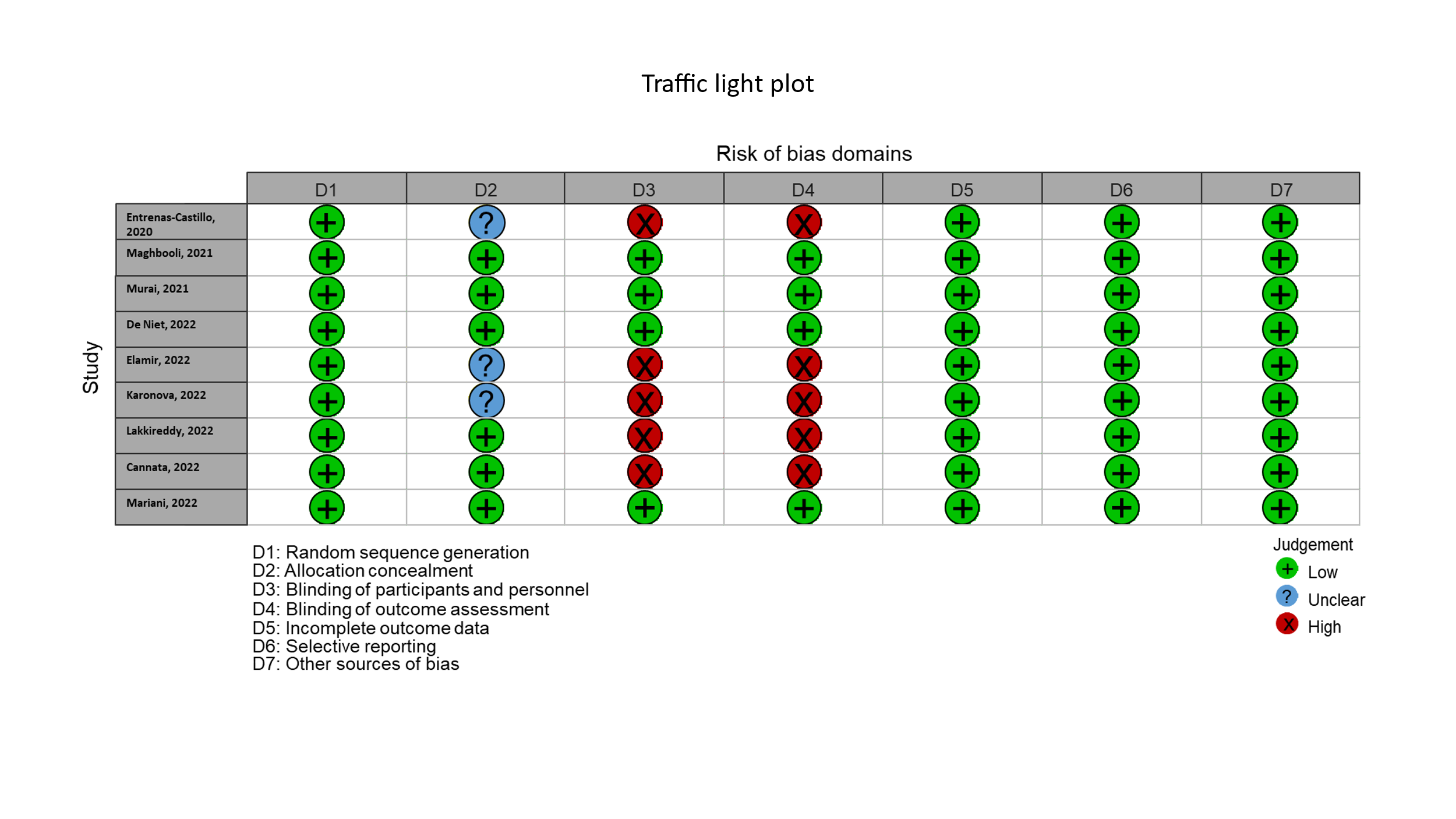

Supplement: S1 Fig — (DOCX) [file pone.0303316.s002.docx]
