# Supplementary material for: Vitamin D, acute respiratory infections, and Covid-19: The curse of small-size randomised trials. A critical review with meta-analysis of randomised trials
Source: PLoS One. 2025 Jan 14;20(1):e0303316. doi: 10.1371/journal.pone.0303316 (PMC11731873; doi:10.1371/journal.pone.0303316)
Supplement: S1 Table — (DOCX) [file pone.0303316.s003.docx]

**S1 Table. Studies identified during the literature search, with inclusions and exclusion**

| **Study** | **Title** | | **Inclusion** | **Exclusion** | **Dose** |
| --- | --- | --- | --- | --- | --- |
| 1/ Entrenas-Castillo, 2020 [35] | Effect of calcifediol treatment and best available therapy versus best available therapy on intensive care unit admission and mortality among patients hospitalized for COVID-19: A pilot randomized clinical study | | consecutive patients hospitalized with COVID-19 infection clinical picture of acute respiratory infection, confirmed by a radiographic pattern of viral pneumonia and by a positive SARS-CoV-2 PCR with CURB65 severity scale  - Age ≥ 18 and < 90 years  - PCR confirmed diagnosis of COVID-19. As an alternative to PCR, a determination of antigens or any other test that in the future is considered equivalent to these in its diagnostic value may be accepted as evidence  - Radiological image compatible with inflammatory pleuropulmonary exudate or patients with onset of symptoms in the last 7 days and with an uncomplicated respiratory infection for outpatient follow-up.  - Signature of direct or delegated informed consent | Patients younger than 18 years and pregnant women were not included  (Serum 25OHD concentrations at baseline or during treatment are not available)  - Being treated with Calcifediol or Cholecalciferol in any of its presentations and dosages  - Intolerance or allergy to Calcifediol or its components  - Pregnancy | oral Calcifediol in soft capsules (0.532 mg) – day 1 + oral calcifediol (0.266 mg) on day 3 and 7, and then weekly until discharge or ICU admission |
| 2/ Maghbooli, 2021 [36] | Treatment With 25-Hydroxyvitamin D(3) (Calcifediol) Is Associated With a Reduction in the Blood Neutrophil-to-Lymphocyte Ratio Marker of Disease Severity in Hospitalized Patients With COVID-19: A Pilot Multicenter, Randomized, Placebo-Controlled, Double-Blinded Clinical Trial. | | 1. Older than 18 years old  2. No medications or disorders that would affect vitamin D metabolism  3. Vitamin D deficiency/  insufficiency (25[OH]D3 concentration of <30 ng/mL)  4. Ability and willingness to give informed consent and comply  with protocol requirements  Treatment With 25-Hydroxyvitamin D3 (Calcifediol) Is Associated  With a Reduction in the Blood Neutrophil-to-Lymphocyte Ratio  Marker of Disease Severity in Hospitalized Patients With COVID-19 | 1. Pregnant or lactating women  2. Severe underlying diseases, such as advanced malignant tumour and end-stage lung disease  3. Chronic hepatic dysfunction and intestinal malabsorption syndromes including inflammatory bowel disease  4. Ongoing treatment with pharmacologic doses of vitamin D, vitamin D metabolites, or analogues  5. Supplementation with over-the-counter formulations of vitamin D2 or vitamin D3  6. Use of tanning bed or artificial ultraviolet exposure within the last 2 weeks  7. Consuming medication affecting vitamin D metabolism or absorption (anticonvulsants, antituberculosis medication glucocorticoids, HIV medications and cholestyramine)  8. History of an adverse reaction to orally administered vitamin D, vitamin D metabolites, or analogues  9. History of an elevated serum calcium concentration of >10.6 mg/dL that was corrected for albumin concentration or subjects with a history of hypercalciuria and kidney stones  10. History of conditions that could lead to high serum calcium concentrations, such as sarcoidosis, tuberculosis, and some lymphomas associated with activated macrophages, which increase the production of 1,25(OH)2D  11. Inability to give informed consent | The dose of 25(OH)D3 was 25 mg administered orally once daily |
| 3/ Murai, 2021 [37] | Effect of a Single High-Dose Vitamin D3 on the Length of Hospital Stay of Severely 25-Hydroxyvitamin D-Deficient Patients with COVID-19 | | age 18 years or older; diagnosis of COVID-19 via PCR testing for SARS-CoV-2 from nasopharyngeal swabs or computed tomography scan findings compatible with the disease (bilateral multifocal ground-glass opacities ≥50%); and diagnosis of flu syndrome with institutional criteria for hospitalization on hospital admission, presenting respiratory rate greater than 24/min, saturation less than 93%while breathing room air, or risk factors for complications (eg, heart disease, diabetes, arterial hypertension, neoplasms,  immunosuppression, pulmonary tuberculosis, obesity) followed byCOVID-19confirmation | unable to read and sign the written informed consent form, were already admitted and receiving invasive mechanical ventilation, received previous vitamin D3 supplementation (>1000 IU/d), had kidney failure requiring dialysis or creatinine of at least 2.0 mg/dL, had hypercalcemia (total calcium >10.5mg/dL),were pregnant or lactating, or had expected hospital discharge in less than 24hours. | single dose of 200.000 IU of vitamin D3 |
| 4/ De Niet, 2022 [38] | Positive Effects of Vitamin D Supplementation in Patients Hospitalized for COVID-19: A Randomized, Double-Blind, Placebo-Controlled Trial | | Caucasian subjects, male and female, aged 18 years or older, with vitamin D deficiency (defined as serum calcifediol concentration < 20 ng/mL) and hospitalized for confirmed SARS-CoV-2 infection at screening were recruited. To be included in the study, the patients were expected to survive for at least 96 h after study entry. | patients presenting acute impairment of renal function or nephrolithiasis. Patients with hypercalcemia and/or hypercalciuria and/or pseudohypoparathyroidism were also excluded at screening. Concomitant medications susceptible to interfere with the study results were not allowed, and subjects who had used any type of vitamin D supplement at screening visit were excluded. | Daily dose of 25,000 international units (IU) vitamin D administered over 4 consecutive days, followed by a weekly dose of 25,000 IU |
| 5/ Elamir, 2022 [39] | A randomized pilot study using calcitriol in hospitalized COVID-19 patients. | | consecutive hospitalized adult patients with COVID-19 | Patients were excluded if they are admitted directly to the intensive care unit (ICU), if they had any of the following: hypercalcemia and/or hyperphosphatemia on admission blood tests, untreated disorders of calcium metabolism including hyperparathyroidism,  hypoparathyroidism, chronic renal insufficiency with glomerular filtration rate < 30 ml/min, or if they are prescribed calcitriol for  any reason outside of the study. | calcitriol 0.5 μg daily for 14 days or hospital discharge |
| 6/ Karonova, 2022 [40] | Effect of Cholecalciferol Supplementation on the Clinical Features and Inflammatory Markers in Hospitalized COVID-19 Patients: A Randomized, Open-Label, Single-Center Study. | | age from 18 to 75 years, confirmed diagnosis of COVID-19 (polymerase chain reaction (PCR)-test and/or chest computed tomography (CT)  scan), and signed informed consent | Subjects with daily vitamin D intake of 1000 IU and higher or who had contraindications to vitamin D supplementation were not included. Additional exclusion criteria were clinically significant kidney pathology with an eGFR of less than 45 mL/min/1.73 m2; gastrointestinal and liver diseases; granulomatous diseases; oncology diseases (less than 5 years); immunodeficiency disorders; and addiction to drugs and alcohol. We did not include pregnant or breastfeeding women. Potential subjects with other circumstances considered inappropriate by the investigator | bolus of cholecalciferol at a dose of 50,000 IU on the 1st and the 8th day of hospitalization, with the total dose being 100,000 IU |
| 7/ Lakkireddy, 2022 [32] | Effect of Short Term High Dose Oral Vitamin D Therapy on the Inflammatory Markers in Patients with COVID 19 Disease. | | Confirmed COVID-19 patients above the age of 18 years with hypovitaminosis D (vit.D level below 30ng/ml) and mild to moderate illness | Patients with severe illness and patients who have taken high dose vit.D (60000 IUs) in the last 3 months, patients with active malignancy, chronic renal disease and HIV, pregnant and breastfeeding mothers were excluded | adjunctive Pulse D therapy (60,000 IUs of vit.D in the form of aqueol nano solution-Deksel® per day for 8 days for  subjects with body mass index (BMI) of 18-25 and 10 days for  subjects with BMI >25) along with the routine standard treatment for COVID-19 |
| 8/ Cannata, 2022 [16] | A single-oral bolus of 100,000 IU of cholecalciferol at hospital admission did not improve outcomes in the COVID-19 disease: the COVID-VIT-D-a randomised multicentre international clinical trial. | | Eligible participants were aged 18 years or above requiring hospitalisation for moderate-severe COVID-19 disease who consented the participation in the study | Patients with dementia or not able to communicate, tested negative for Severe Acute Respiratory Syndrome Coronavirus 2 (SARS-CoV-2) despite clinical findings compatible with COVID-19 disease, pregnant and lactating women, patients who received any form of  vitamin D in the previous 3 months and allergic to vitamin D | single oral bolus of 100,000 IU of cholecalciferol administered at hospital admission |
| 9/ Mariani, 2022 [41] | High-dose vitamin D versus placebo to prevent complications in COVID-19 patients: Multicentre randomized controlled clinical trial. | | adults aged 18 or older patients and either gender, who had been admitted to general wards in the last 24 hours, with SARS-CoV-2 confirmed infection by reverse transcriptase–polymerase chain reaction, an expected hospitalization for at least 24 hours, oxygen saturation  >90% (measured by pulse oximetry) breathing ambient air, and at least one of the following  conditions: age 45 or older or hypertension, diabetes, chronic obstructive pulmonary disease or  asthma (at least moderate), cardiovascular disease (history of myocardial infarction, percutaneous  transluminal coronary angioplasty, coronary artery bypass grafting or valve replacement surgery)  or body mass index >30 (S1 File). Age 45 or older was selected as inclusion criterion | Main exclusion criteria were >72  hours since admission, women in childbearing age, requirement for >5 litres/minute of oxygen or mechanical ventilation, chronic kidney disease requiring haemodialysis or chronic liver failure, chronic supplementation with pharmacological vitamin D, treatment with anticonvulsants, sarcoidosis, malabsorption syndrome, known hypercalcemia, life expectancy <6 months, allergy to study medication, or any condition at discretion of investigator impeding to understand the study and give informed consent | single oral dose of 500 000 IU of vitamin D3 soft gel capsules  (5 capsules of 100 000 IU) |
|  | | **Excluded from the meta-analysis** | | | |
| 1/ Jolliffe, 2022 [14] | Jolliffe DA, Holt H, Greenig M, Talaei M, Perdek N, Pfeffer P, et al. Effect of a test-and-treat approach to vitamin D supplementation on risk of all cause acute respiratory tract infection and covid-19: phase 3 randomised controlled trial (CORONAVIT) | | current residence in the UK, age 16 years or older at screening, enrolment in COVIDENCE UK, and online provision of informed consent | Admission to ICU not a specific outcome  **Taking vitamin D supplements,** digoxin, alfacalcidol, calcitriol, dihydrotachysterol or paricalcitol; diagnosis of sarcoidosis, primary hyperparathyroidism, nephrolithiasis, or renal failure requiring dialysis; allergy to any ingredient in the study capsules; and pregnancy. | supply of 800 IU/day vitamin D supplements if blood  25-hydroxyvitamin D (25(OH)D) concentrations were <75 nmol/L (lower dose group) or 3200 IU/day if blood  concentrations were <75 nmol/L (higher dose group). |
| 2/ Brunvoll, 2022 [15) | Prevention of covid-19 and other acute respiratory infections with cod liver oil supplementation, a low dose vitamin D supplement: quadruple blinded, randomised placebo controlled trial. | | adults (aged ≥18 years) with a Norwegian personal identity number and electronic access to the secure national digital governmental identification service, | Admission to ICU not a specific outcome  Excluded from analysis:  Positive SARS-CoV-2 test  before randomisation ; Positive SARS-CoV-2 test in first 7 days of supplementation | 5 mL of oil contained about  10 μg of vitamin D3 (400 IU) |
| 3/ Sanchez-Zuno, 2021 [34] | Vitamin D Levels in COVID-19 Outpatients from Western Mexico: Clinical Correlation and Effect of Its Supplementation. | |  | Admission to ICU not a specific outcome |  |
| 4/ Samaha, 2022 [44] | Serum 25-hydroxyvitamin D Concentration Significantly Decreases in Patients with COVID-19 Pneumonia during the First 48 Hours after Hospital Admission | |  | Admission to ICU not a specific outcome |  |
| 5/ Villasis-Keever, 2022 [45] | Efficacy and Safety of Vitamin D Supplementation to Prevent COVID-19 in Frontline Healthcare Workers. A Randomized Clinical Trial | |  | Admission to ICU not a specific outcome |  |
| 6/ Abroug, 2023 [46] | Effect of vitamin D supplementation versus placebo on recovery delay among COVID-19 Tunisian patients: a randomized-controlled clinical trial | |  | Admission to ICU not a specific outcome |  |
| 7/ Bishop, 2023 [47] | REsCue trial: Randomized controlled clinical trial with extended-release calcifediol in symptomatic COVID-19 outpatients | |  | Admission to ICU not a specific outcome |  |
| 8/ Soliman, 2021 [33] | Impact of Vitamin D Therapy on the Progress COVID-19: Six Weeks Follow-Up Study of Vitamin D Deficient Elderly Diabetes Patients. | |  | Failure of randomisation |  |
| 9/ Jevalikar, 2021 [48] | Lack of association of baseline 25-hydroxyvitamin D levels with disease severity and mortality in Indian patients hospitalized for COVID-19 | |  | Not a randomised trial |  |
| 10/ Annweiler, 2002 [42] | High-dose versus standard-dose vitamin D supplementation in older adults with COVID-19 (COVIT-TRIAL): A multicenter, open-label, randomized controlled superiority trial. | |  | High vs. low VDS |  |
| 11/ Sabico, 2021 [43] | Effects of a 2-Week 5000 IU versus 1000 IU Vitamin D3 Supplementation on Recovery of Symptoms in Patients with Mild to Moderate Covid-19: A Randomized Clinical Trial | |  | High vs. low VDS |  |

VDS: vitamin D supplementation
